# An estimation of the long-term clinical and economic benefits of insulin lispro in Type 1 diabetes in the UK

**DOI:** 10.1111/j.1464-5491.2009.02775.x

**Published:** 2009-08

**Authors:** C Pratoomsoot, H T Smith, A Kalsekar, K S Boye, J Arellano, W J Valentine

**Affiliations:** IMS HealthAllschwil, Switzerland; * Eli Lilly and CompanySurrey, UK; † Eli Lilly and Company, IndianapolisIN, USA; ‡ Ossian Health Economics and CommunicationsBasel, Switzerland

**Keywords:** cost-effectiveness analysis, insulin lispro, regular human insulin, Type 1 diabetes, UK

## Abstract

**Aims:**

To determine the long-term health economic benefits associated with lispro vs. regular human insulin (RHI) in UK Type 1 diabetic (T1DM) patients using the previously published and validated CORE Diabetes Model.

**Methods:**

A literature review designed to capture clinical benefits associated with lispro and T1DM cohort characteristics specific to UK was undertaken. Clinical benefits were derived from a Cochrane meta-analysis. The estimated difference (weighted mean) in glycated haemoglobin (HbA_1c_) was −0.1% (95% confidence interval −0.2 to 0.0%) for lispro vs. RHI. Severe hypoglycaemia rates for lispro and RHI were 21.8 and 46.1 events per 100 patient years, respectively. Costs and disutilities were accounted for severe hypoglycaemia rates. All costs were accounted in 2007 £UK from a National Health Service (NHS) perspective. Future costs and clinical benefits were discounted at 3.5% annually.

**Results:**

In the base-case analysis, lispro was projected to be dominant compared with RHI. Lispro was associated with improvements in quality-adjusted life expectancy (QALE) of approximately 0.10 quality-adjusted life years (QALYs) vs. RHI (7.60 vs. 7.50 QALYs). Lifetime direct medical costs per patient were lower with lispro treatment, £70 576 vs. £72 529. Severe hypoglycaemia rates were the key driver in terms of differences in QALE and lifetime costs. Sensitivity analyses with assumptions around time horizon, discounting rates and benefits in terms of glycaemic control or hypoglycaemic event rates revealed that lispro remained dominant.

**Conclusions:**

Our findings suggest that lispro is likely to improve QALE, reduce frequency of diabetes-related complications and lifetime medical costs compared with RHI.

## Introduction

Type 1 diabetic (T1DM) patients treated with unmodified regular human insulin (RHI) rarely achieve their glycaemic target and often suffer from postprandial hyperglycaemic excursions, together with an increased risk of hypoglycaemia in the post-absorptive period [1]. The shortcomings of RHI lie with its non-optimal pharmacokinetics which cannot mimic the physiological insulin pattern of non-diabetic individuals and needs to be administered 30–60 min prior to meals [2]. Evidence from randomized open-label trials suggests that rapid/short-acting insulin analogues (SAIAs), such as insulin lispro, are more effective than the conventional RHI in terms of reduced postprandial plasma glucose excursions [1,3–8] and reductions in the frequency of severe and nocturnal hypoglycaemia [9–11] as a result of improved pharmacokinetics [12].

The UK’s National Institute for Health and Clinical Excellence (NICE) has not published full guidance on the use of SAIAs in T1DM patients. The current body of evidence [14–17] suggests that there are clinical benefits associated with the use of SAIAs and these insulins are playing an increasing role in the treatment of T1DM in the UK, as reflected in a 10.8% increase in prescriptions from 2005 to 2006 [13]. Benefits of SAIAs in terms of increased lifestyle flexibility were recognized in a published clinical guideline from NICE on the diagnosis and management of T1DM [14]. In addition, it was recommended that SAIAs should be used as an alternative to mealtime RHI in patients who experience problems with nocturnal or late inter-prandial hypoglycaemia and in those whose blood glucose control does not require the use of snacks between meals.

In recent years, SAIAs have received a considerable amount of attention and there has been a number of publications with regards to their clinical effects and economic implications [15–19], including health technology assessments which have demonstrated that treatment with lispro or aspart significantly reduced glycated haemoglobin (HbA_1c_) compared with RHI in T1DM patients [16,17]. Funding of SAIAs would be expected to increase healthcare budgets [16]. Evidence suggests that the additional costs incurred by SAIAs will be offset by other healthcare costs in the first 12 months [16]. However, there are uncertainties surrounding the long-term effects of SAIAs on complications, mortality and HbA_1c_ because of the lack of high-quality long-term studies [19]. Furthermore, the long-term economic impact over patient lifetimes is unknown. There is a need for additional data on the long-term clinical and economic outcomes associated with the use of SAIAs and RHI in patients with T1DM.

The aim of this study was twofold: first to review the available literature to identify clinical efficacy of SAIAs and published cohort characteristics representative of T1DM patients within the UK. Second, to estimate the long-term clinical and economic outcomes associated with lispro compared with RHI using the previously published CORE Diabetes Model [20], which provides estimates of long-term clinical and cost outcomes that closely match real-life data [21].

## Patients and methods

### Literature review

Electronic searches for clinical efficacy of SAIAs compared with RHI and cohort characteristics representative of T1DM patients within the UK were conducted. Searches were limited to studies specific to humans, published in the English language and between the years of 1990–2008 in the following databases: PubMed, embase and Ovid medline. Combinations of descriptors and keywords were used and searches were performed according to the strategies outlined in the Supporting Information (Appendix S1). Published articles were screened based on titles, keywords and abstract. Potentially relevant articles were then subjected to full-text review. Additional references cited by the articles were obtained where appropriate.

### CORE Diabetes Model

The CORE Diabetes Model, together with its structure and data input interfaces, has been described in detail elsewhere [20]; however, a brief summary is given here. The model projects long-term health and economic outcomes of a cohort of diabetic patients. It takes into account baseline cohort characteristics, history of complications, current and future management of diabetes and concomitant medications, treatment effects and changes in physiological parameters over time. The model is based on a series of sub-models simulating major complications of diabetes such as angina, myocardial infarction, heart failure, peripheral vascular disease, stroke, neuropathy, foot ulcer, amputation, renal disease and eye disease. Each sub-model is a Markov model using Monte Carlo simulation incorporating time, state, time-in state and transition probabilities derived from published sources. Output data in terms of development of complications, life expectancy, quality-adjusted life expectancy (QALE), incremental cost-effectiveness ratio (ICER), annual costs per patient and cumulative costs per patient can be projected. The model allows for analyses specific to type of diabetes, cohorts, countries and interventions. Thus, investigation around new interventions can be made and comparison between management strategies in realistic clinical settings can be achieved. The outcomes simulated by the model have been validated previously against other published epidemiological and clinical studies [21].

### Simulation cohort

Baseline cohort characteristics representative of T1DM patients in the UK were derived from several published studies based on the results of the literature review [22–31]. Patient demographics, baseline complications and medical history were sourced from records of primary care physicians in the UK, The Health Improvement Network (THIN) database [22]. This includes records for over 2.3 million active patients and is considered to be representative of the UK population (and the UK population with T1DM). Baseline risk factors such as HbA_1c_, systolic blood pressure, lipoproteins and triglyceride levels were derived from T1DM patient records, who attended the diabetes services in Newcastle upon Tyne, where the data were collected prospectively over a 9-year period [25]. Racial characteristics by ethnic group within the UK population were obtained from the Office for National Statistics UK [32]. Long-term clinical and economic outcomes were calculated using a simulated population based upon the baseline demographics, complications and use of concomitant medications. Baseline cohort characteristics and complications are given in detail in Table 1.

**Table 1:** Baseline characteristics, complications, concomitant medications and management of patients in the simulated cohort

Patient demographics	Mean	sd	References
Sex (% male)	53.4	—	[22]
Mean age (years)	37.8	—	[22]
Duration of diabetes (years)	10.4	—	[22]
BMI (kg/m^2^)	25.6	—	[22]
Ethnic origin (%)			
Caucasian	93.5	—	[32]
Black	2	—	[32]
Hispanic	0	—	[32]
Native American	0	—	[32]
Asian	4.5	—	[32]
Risk factors			
Glycated haemoglobin (HbA_1c_) (%)	9.4	2.10	[25]
Systolic blood pressure (mmHg)	132	21.00	[25]
Total cholesterol (mmol/l)	5.4	—	[25]
High-density lipoproteincholesterol (mmol/l)	1.5	—	[25]
Low-density lipoproteincholesterol (mmol/l)	3.2	—	[25]
Triglycerides (mmol/l)	1.2	—	[25]
Proportion of smoker (%)	16.4	—	[25]
Pre-existing complications			
Myocardial infarction (%)	1.7		[22]
Peripheral vascular disease (%)	1.6		[22]
Stroke (%)	2.2		[22]
Heart failure (%)	0.5		[22]
Microalbuminuria (%)	20		[25]
Background diabeticretinopathy (%)	27.46		[25]
Neuropathy (%)	9.88		[25]
Patient management			
Taking aspirin (%)	4.3		[26]
Taking statins (%)	17.8		[27]
Taking ACE-I (%)	14.6		[28,29]
Screened for retinopathy (%)	63.2		[31]
Screened for renal disease (%)	60		[30]

ACE-I, angiotensin converting enzyme inhibitor; BMI, body mass index; sd, standard deviation.

### Intervention effects

Clinical effects of lispro and RHI were derived from the results of a meta-analysis (the Cochrane review) [17], which examined the effects of SAIAs vs. RHI. It reported an estimated difference (weighted mean) (WMD) in HbA_1c_ of −0.1% [95% confidence interval (CI) −0.2 to 0.0] in favour of lispro in comparison with RHI. In the lispro arm of the base-case simulation, an HbA_1c_ benefit of −0.1% was applied to the baseline HbA_1c_ of 9.4% and simulated over patient lifetimes. Significant heterogeneity between trials within the meta-analysis was observed (*P* = 0.02) [17]. Lispro-specific hypoglycaemic event rates were not reported in the Cochrane meta-analysis and therefore the reported hypoglycaemic event rates for SAIAs in general were used instead. Severe hypoglycaemia episodes were defined as those requiring third party assistance. However, it was found that, for the studies included in the Cochrane systematic review, the definitions of severe hypoglycaemia ranged from third-party help to coma and/or use of glucagon or glucose. Of the 28 included studies, the incidence of severe hypoglycaemia ranged from 0 to 247.3 (median 21.8) episodes per 100 patient years for SAIAs and from 0 to 544 (median 46.1) for patients treated with RHI. Median values were computed by dividing the number of severe hypoglycaemic episodes by the years of exposure and then multiplying by 100.

The Cochrane systematic review also included the analysis of overall hypoglycaemic episodes for patients with T1DM treated with SAIAs vs. RHI. Ten studies were included; however, heterogeneity between the included studies was acknowledged where definitions of hypoglycaemia ranged from less than 2.0 mmol/l to < 3.9 mmol/l with or without symptoms.

In the modelling analysis, minor hypoglycaemia was defined as events not requiring third-party assistance or hospital admission. The rates of minor hypoglycaemia were derived indirectly by calculating the overall hypoglycaemia rates (mean events per 100 patient years) from the 10 included studies (weighted by the number of patients in each study). Subsequently, the reported severe hypoglycaemic event rates were subtracted from the overall rates to arrive at the minor hypoglycaemia rates of 6790 and 7311 events per 100 patient years for analogues vs. regular human insulin, respectively.

For the base-case modelling analysis, the severe hypoglycaemic event rates of 21.8 events/100 patient years vs. 46.1 events/100 patient years were used for SAIAs and RHI, respectively. A conservative approach was taken and minor hypoglycaemic event rates were not included in the base-case analysis.

Patients were assumed to remain on the same treatment regimens throughout the simulation. After the initial benefit (−0.1%) was applied in the lispro arm, HbA_1c_ was assumed to follow a progression in both arms based on data from the Diabetes Control and Complications Trial (DCCT) [34]. In the absence of long-term data, hypoglycaemic event rates in both treatment groups were assumed to remain constant over the course of the simulation. It was also conservatively assumed that severe hypoglycaemic events did not result in fatalities in T1DM, but affected the quality of life (as a result of event disutilities) and costs.

Cohort parameters of typical T1DM patients in the UK, such as age, duration of diabetes, baseline HbA_1c_ and complications, were sourced from cross-sectional and observational studies and regional data as opposed to randomized controlled studies. In this manner, bias relating to patient selection in randomized studies is minimized. The rationale for using HbA_1c_ benefits from a meta-analysis was that clinical effects of SAIAs were drawn from studies with different designs and different patient characteristics. This would avoid any bias from one particular study.

### Costs

Current prices of insulin lispro (Humalog), RHI (Humulin R) and basal neutral protamine Hagedorn (NPH) insulin (Humulin I) were obtained from the Monthly Index of Medical Specialties (MIMS) [35]. The costs of insulins were based on weighted averages of the main insulin products. For the base-case, the annual costs of insulins were calculated based on the reported end-of-trial doses in a study of patients with a diagnosis of T1DM for more than 2 years on established basal–bolus regimens aiming for tight glucose control, comparing two treatment arms, insulin lispro plus basal NPH insulin [32.25 plus 20.25 International Units (IU)] vs. RHI plus basal NPH insulin (32.25 plus 20.25 IU) in the UK [33]. The annual costs of treatment were estimated to be £786.83 in the lispro arm and £775.44 in the RHI arm. Costs associated with self-monitoring of blood glucose (SMBG) were also included. Diabetes and UK-specific direct medical costs incorporating pharmacy costs and costs of complications were derived from published sources [36–44] (see also Supporting Information, Appendix S2). All costs were expressed in 2007 £UK. Where costs were taken from earlier published literature, they were inflated to 2007 values using the appropriate UK consumer price indices, accounted from a third-party healthcare payer, National Health Service (NHS) perspective and hence indirect costs were not considered.

### Health state utilities

Estimates of health-related quality of life utilities of patients with diabetes (utility weights that are used to represent preferences for health states) corresponding to myocardial infarction, ischaemic heart disease, stroke, heart failure, amputation and blindness were derived from the UK Prospective Diabetes Study (UKPDS) [45]. Other utilities were supplemented by other published sources [46–50].

### Discounting and time horizon

Future costs and health benefits were discounted at a rate of 3.5%*per annum* as recommended by NICE, UK [51]. A time horizon of 50 years was used in the base-case analysis. The simulations aimed to capture death of all patients in the simulated cohort within 50 years and to project long-term complications with their associated costs and consequently the impact on life expectancy and quality of life over patient lifetimes.

### Sensitivity analyses

Sensitivity analyses were performed around the assumptions in the base-case analysis. Key parameters were varied over a range of possible scenarios, assessing their impact on health economic outcomes. We investigated the impact of the time horizon by varying the time between 0 and 30 years (here we report values at 5, 10, 15, 20, 25 and 30 years). Discount rates for costs and health outcomes were applied at 0 and 7%*per annum*. The impact of changes in HbA_1c_ on long-term clinical and economic benefits was assessed by applying no change in HbA_1c_ and −0.2% change for the insulin lispro arm (in line with the upper and lower 95% CI from the Cochrane meta-analysis [17]), which spans the range of values reported in another recent meta-analysis published by the Canadian Agency for Drugs and Technologies in Health, WMD −0.09%, 95% CI −0.16 to −0.01 [16]. The impact of a lower baseline HbA_1c_ of 6.3%, derived from a UK-specific lispro study [33], on the long-term clinical and economic outcomes was also assessed. The influence of hypoglycaemic event rates was determined. In one sensitivity analysis, severe hypoglycaemic event rates of lispro were applied to both treatment arms (i.e. assumed no difference). In another sensitivity analysis, minor hypoglycaemic event rates were included in addition to severe hypoglycaemia rates. The lispro regimen was associated with 6790.13 events/100 patient years in comparison with 7311.75 events/100 patient years for RHI (difference of 521.62 events/100 patient years). To assess the impact of insulin dose on the economic outcomes, the same treatment effects as in the base-case were assumed, but treatment costs were analysed based on varying dosages. First, it was assumed that patients received 54 IU/day for each of the insulin treatments; i.e. 54 IU/day for prandial insulin plus 54 IU/day for basal insulin (total 108 IU/day for each treatment arm). Second, a conservative approach from a modelling perspective was assumed, the insulin lispro dose was increased to 54 IU/day and all other insulins remained the same as in the base-case. Third, it was assumed that there were dosage benefits associated with lispro treatment, thus RHI was set to 54 IU/day and all other insulins remained the same as those of the base-case.

### Statistical methodology

For each analysis in the base-case and sensitivity analyses, 1000 × 1000 iterations were performed based on the simulation cohort. Using a non-parametric bootstrapping approach, 1000 mean costs and effect pairs (each of 1000 iterations) were calculated for each treatment group [52]. The joint density of mean incremental costs and incremental effectiveness (in terms of quality-adjusted life expectancy) for lispro vs. RHI were plotted as a scatter plot on a cost-effectiveness plane. The uncertainty surrounding the cost-effectiveness of lispro vs. RHI was assessed through a range of willingness-to-pay thresholds. From this, cost-effectiveness acceptability curves were generated for the base-case and the sensitivity analysis assessing the impact of severe hypoglycaemia.

## Results

### Literature review

Cohort characteristics typical of T1DM adult patients in the UK resulting from the literature search are defined in Patients and methods.

#### Treatment effects of short-acting insulin analogues

From the literature search 2284 articles were identified. Of these, 2232 articles were excluded because they were reviews, pharmacodynamic or pharmacokinetic studies, non-randomized controlled trials, studies of Type 2 and gestational diabetes or trials with less than 4 weeks’ study period. From 52 potentially relevant articles, six studies were excluded because they were of diabetes in infants and young patients. Forty-four studies were selected [1,3,4,6–8,10,33,53–88], reporting the clinical benefits associated with the treatment of SAIAs in T1DM patients. We also identified two meta-analyses [16,17] containing relevant data for the inclusion in the literature review and the cost-effectiveness analysis. They reported clinical outcomes associated with SAIAs in comparison with RHI in patients with Type 1, Type 2 and gestational diabetes. Their outcome measures were HbA_1c_, blood glucose levels, hypoglycaemia, adverse events, mortality and quality of life.

Of the 44 studies identified, 29 showed benefits in terms of HbA_1c_ reductions from baseline associated with SAIAs compared with RHI [1,3,6,33,53,55,58–61,63,67,69–77,79,82–88]. Thirteen of these 29 estimated differences in favour of SAIAs that were statistically significant at the 5% level [3,6,53,55,67,69,71,75,82,83,85,86,88]. The magnitude of differences in HbA_1c_ between SAIAs and RHI was relatively small; mean difference between treatments in HbA_1c_ reductions ranged from −0.01 to −0.77%. Fourteen studies demonstrated that SAIAs compared with RHI resulted in improved postprandial glucose excursion control, but had no effect on HbA_1c_ or were not associated with significant benefits in reducing HbA_1c_ [4,7,8,10,54,56,57,62,64–66,78,80,81]; one study showed significantly higher treatment satisfaction and treatment flexibility scores for T1DM treated with lispro vs. RHI [68].

Twenty-three studies demonstrated that SAIAs were associated with lower hypoglycaemia rates [3,4,7,8,10,33,53,55,60,63,64,71,73–75,77,79–81,85–88]. There were mean differences in endpoint hypoglycaemic event rates (not all statistically significant), which ranged from −0.1 to −4.1 episodes per patient per month in favour of SAIAs when compared with RHI. Studies specific to lispro demonstrated that injections of lispro immediately before meals lowered postprandial serum glucose excursions compared with patients treated with RHI [4,8]. Furthermore, treatment with lispro resulted in lower hypoglycaemic event rates, with the largest improvement during night-time [4,8,33]. Importantly, patients with T1DM treated with lispro were also reported to have achieved significantly lower HbA_1c_ [53]. Clinical benefits were more pronounced with the use of continuous subcutaneous insulin infusion (CSII) [6,67,69,71,73,75]; fluctuations in postprandial blood glucose levels were significantly reduced. HbA_1c_ was significantly lower and the insulin requirement was slightly but significantly lower with lispro.

Two recent meta-analyses [16,17] gave accounts of the clinical effectiveness of SAIAs vs. RHI. The Cochrane review [17] published findings based on meta-analysis performed on randomized controlled trials with an intervention duration of at least 4 weeks. The reviewers identified 49 potential randomized controlled trials, but excluded 24 studies for reasons such as the absence of baseline HbA_1c_ or follow-up data, studies performed on pre-pubertal children, adolescents and women with gestational diabetes. Sixteen studies compared lispro vs. RHI; the HbA_1c_ change from baseline was greater with lispro than RHI in T1DM patients. The WMD of HbA_1c_ was −0.1% (95% CI −0.2 to 0.0) in favour of lispro. Subgroup analyses of studies of different types of interventions suggest that using CSII was more effective compared with intensive insulin therapy (IIT). The WMD was −0.2% (95% CI −0.3 to −0.1) comparing insulin analogues to RHI. Furthermore, SAIAs were associated with greater benefits in terms of severe and minor hypoglycaemic event rates.

Findings from the Cochrane review are supported by a second meta-analysis published by the Canadian Agency for Drugs and Technologies in Health (CADTH) [16]. The analysis included 47 studies on T1DM, 34 of which described the use of lispro, in which significantly greater reductions in HbA_1c_ levels with lispro compared with RHI were reported. The WMD was −0.09% (95% CI −0.16 to −0.01). In addition, the difference was more pronounced in patients receiving CSII; WMD was −0.28% (95% CI −0.45 to −0.12). However, the overall and severe hypoglycaemic event rates were similar with the two treatments, but the occurrence of nocturnal hypoglycaemia was less frequent with lispro in comparison with RHI.

The WMD of HbA_1c_ from the Cochrane meta-analysis was similar to that of the CADTH meta-analysis (−0.1 and −0.09%, respectively); the upper and lower 95% CI from the Cochrane meta-analysis also spans those reported in the CADTH meta-analysis (−0.2 to −0.0 and −0.16 to −0.01, respectively). For these reasons, treatment effects, WMD of HbA_1c_, from the Cochrane meta-analysis were employed in our base-case and sensitivity analyses.

### CORE Diabetes Model simulation

#### Long-term clinical outcomes

Long-term projections of treatment with lispro vs. RHI in a ‘typical’ UK T1DM cohort and based on treatment effects from the Cochrane meta-analysis indicated that treatment with insulin lispro was associated with improvements in life expectancy and QALE (discounted by 3.5%*per annum*). In the base-case simulation, mean discounted life expectancy increased by 0.06 years and the mean QALE increased by 0.105 quality-adjusted life years (QALYs) with lispro compared with RHI (Table 2). Higher severe hypoglycaemic event rates in the RHI arm had a notable impact on patients’ quality of life.

**Table 2:** Summary of base-case analysis: clinical and economic outcomes of treatments with insulin lispro vs. regular insulin

Description of outcome	Lispro	Regular insulin	Difference
Life expectancy (years)	11.90 (0.179)	11.844 (0.167)	0.06
Quality-adjusted life expectancy (years)	7.601 (0.117)	7.497 (0.107)	0.105
Lifetime direct medical costs (£)	70 576 (1774)	72 529 (1793)	−1953
ICER based on life expectancy		Dominant	
ICER based on quality-adjusted life expectancy		Dominant	

ICER, incremental cost-effectiveness ratio.

Values shown are means with standard deviation in parentheses.

All costs and clinical outcomes are discounted at 3.5%*per annum*.

The cumulative incidence of diabetes-related complications such as eye disease, renal complications and cardiovascular diseases (CVDs) were projected to be lower during treatment with lispro in comparison with RHI (Table 3). In addition, lispro was projected to delay time of onset of most diabetes-related complications (Table 4). The mean time to onset of any diabetes-related complication was 0.45 years for lispro and 0.43 years for RHI (an estimated difference of 7.3 days).

**Table 4:** Summary of the mean time to onset of complications

	Time to onset of complications (years)		
Complication	Lispro	Regular insulin	Difference
Any complications	0.45	0.43	0.02
Background retinopathy	3.01	2.86	0.15
Proliferative retinopathy	12.24	12.05	0.19
Microalbuminuria	5.02	4.93	0.09
Gross proteinuria	8.27	8.08	0.19
End-stage renal disease	15.69	15.58	0.11
First event ulcer	13.63	13.49	0.14
Amputation	16.26	16.13	0.13
Neuropathy	3.46	3.34	0.12
Peripheral vascular disease	15.98	15.83	0.15
Congestive heart failure	15.98	15.87	0.11
Angina	16.44	16.36	0.08
Myocardial infarction	15.92	15.78	0.14
Stroke	16.79	16.68	0.11
Cataract	15.99	15.88	0.11
Macular oedema	12.79	12.62	0.17
Severe vision loss	14.60	14.47	0.13

Time to onset of diabetes-related complications of the base-case.

Values expressed are means.

**Table 3:** Cumulative incidence of diabetes-related complications and adverse events of base-case analysis

	Cumulative incidence diabetes-related complications (%)		
Complication	Lispro	Regular insulin	Difference
Background diabetic retinopathy	83.16 (1.50)	83.92 (1.42)	−0.76
Proliferative diabetic retinopathy	32.11 (1.49)	33.28 (1.46)	−1.17
Macular oedema	39.67 (1.57)	40.10 (1.53)	−0.43
Severe vision loss	25.30 (1.35)	25.76 (1.34)	−0.46
Cataract	12.11 (1.02)	12.00 (1.02)	0.11
Microalbuminuria	75.50 (1.91)	75.83 (2.05)	−0.33
Gross proteinuria	67.67 (1.76)	68.45 (1.91)	−0.78
End-stage renal disease	30.76 (1.61)	30.95 (1.62)	−0.19
Nephropathy-related death	28.48 (1.48)	28.64 (1.46)	−0.16
Ulcer	47.12 (1.57)	47.19 (1.55)	−0.07
Recurrent ulcer	65.37 (4.29)	66.01 (4.46)	−0.64
Amputation	14.25 (1.33)	14.35 (1.23)	−0.1
Recurrent amputation	7.09 (1.17)	7.03 (1.04)	0.06
Neuropathy	89.30 (1.03)	89.52 (1.02)	−0.22
Coronary heart failure death	9.99 (0.97)	9.80 (0.92)	0.19
Coronary heart failure event	23.84 (1.46)	23.65 (1.33)	0.19
Peripheral vascular disease	15.90 (1.08)	16.31 (1.14)	−0.41
Angina	8.61 (0.91)	8.40 (0.87)	0.21
Stroke death	3.82 (0.63)	3.85 (0.63)	−0.03
Stroke event	8.20 (0.89)	8.14 (0.86)	0.06
Myocardial infarction death	20.00 (1.29)	20.18 (1.28)	−0.18
Myocardial infarction event	31.70 (1.45)	32.09 (1.47)	−0.39
Severe hypoglycaemia	7.59 (0.18)	14.20 (0.32)	−6.61

Incidence expressed as a mean percentage with standard deviation in parentheses.

#### Long-term economic outcomes

Treatment with lispro was associated with lower direct medical costs over patients’ lifetimes compared with RHI (£70 576 vs. £72 529 per patient, respectively). The breakdown of costs demonstrated that the key driver for the difference in direct medical costs (£1953 per patient) was the higher severe hypoglycaemic event rates in the RHI arm (Table 5). Overall costs of complications during patient lifetimes were marginally lower in lispro for CVD and eye and foot complications. Lispro was projected to be a dominant intervention to RHI.

**Table 5:** Breakdown of lifetime direct medical costs per patient

	Breakdown of direct costs (£)		
Description of cost	Lispro	Regular insulin	Difference
Total costs	70 576	72 529	−1953
Treatment costs	9810	9623	187
Management costs	1375	1372	3
Cardiovascular disease costs	5645	5695	−50
Renal disease costs	26 912	26 844	68
Diabetic foot and neuropathy costs	22 542	22 714	−172
Eye disease costs	2034	2048	−14
Hypoglycaemia costs	2258	4233	−1975

Breakdown of total lifetime costs per patient of the base-case; values shown are means.

**FIGURE 1:**
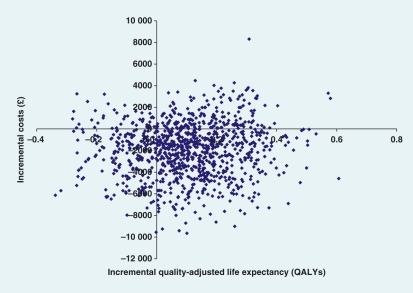
Incremental cost-effectiveness ratio scatter plot for lispro vs. regular insulin. Base-case analysis incremental cost-effectiveness ratio scatter plot of 1000 values of mean incremental costs plotted against mean incremental effectiveness (quality-adjusted life years gained). The scatter plot was generated for Type 1 diabetes patients treated with a basal–bolus regimen of lispro vs. regular human insulin. The majority of incremental cost–effect pairs lie in the south-east quadrant, indicating dominance for lispro vs. regular human insulin, where lispro was projected to be more effective and cost saving.

**FIGURE 2:**
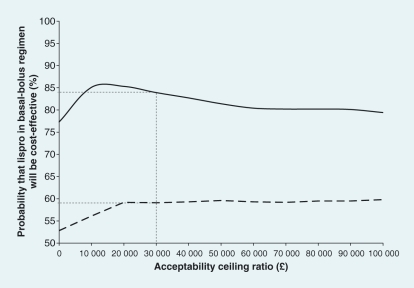
Cost-effectiveness acceptability curves for lispro vs. regular human insulin. Curve in solid line shows cost-effectiveness acceptability curve for basal–bolus regimens of lispro vs. regular human insulin for the base-case analysis. The acceptability curve demonstrates the likelihood of lispro being considered cost-effective for a range of acceptable ceiling ratios. There is a probability of 83.9% that lispro will be cost-effective compared with regular human insulin at a threshold of £30 000. In a univariate sensitivity analysis where severe hypoglycaemia rates for both treatment arms were assumed to be identical (curve in dashed line), the resulting curve demonstrates that there is a 59.1% probability that lispro will be cost-effective compared with regular human insulin at a threshold of £30 000.

An incremental cost-effectiveness scatter plot was generated by plotting the 1000 mean costs and effect pairs (QALE) for lispro vs. RHI (Fig. 1). The figure shows that most points were in the south-east quadrant of the plane, indicating the dominant nature of lispro (increased effectiveness and lower overall costs). The likelihood of lispro being considered cost-effective was determined for a range of acceptability ratios. For the base-case scenario, there was a probability of 83.9% that lispro will be cost-effective at a threshold of £30 000 (Fig. 2, solid curve).

### Sensitivity analyses

Sensitivity analyses revealed that results of the simulation were most sensitive to changes in hypoglycaemic event rates (Table 6). When no difference in severe hypoglycaemia rates was applied, lispro was associated with a benefit in terms of mean quality-adjusted life expectancy of approximately 0.034 QALYs vs. RHI, compared with a benefit of 0.105 QALYs in the base-case. Cost savings with lispro were also reduced. The mean saving over a patient’s lifetime was approximately £173, assuming no difference in severe hypoglycaemia (compared with £1953 in the base-case). The uncertainty of lispro being considered cost-effective at a threshold of £30 000 was also demonstrated in the cost-effectiveness acceptability curve for this scenario (Fig. 2, dashed curve). When benefit in severe hypoglycaemia associated with lispro was abolished, the resulting probability that lispro will be cost-effective was 59.1%. Accordingly, there was an uncertainty of 40.9% that lispro will not be cost-effective. Capturing minor hypoglycaemic events in the analysis notably increased the improvement in quality-adjusted life expectancy associated with lispro. In this scenario, lispro treatment was projected to improve mean quality-adjusted life expectancy by approximately 0.355 QALYs vs. RHI. As minor hypoglycaemic events were conservatively assumed not to incur costs from a healthcare payer perspective, capturing minor hypoglycaemic events in the simulation did not alter lifetime direct costs.

**Table 6:** Summary of sensitivity analyses comparing lispro vs. regular insulin

	Quality-adjusted life expectancy (QALYs)			Lifetime direct costs per patient (£)			
Assumption	Lispro	Regular insulin	Difference	Lispro	Regular insulin	Difference	ICER/£ per QALY gained
Base-case	7.601 (0.117)	7.497 (0.107)	0.105 (0.154)	70 576 (1774)	72 529 (1793)	−1953 (2508)	Dominant
5-year time horizon	2.909 (0.024)	2.885 (0.025)	0.025 (0.035)	19 562 (940)	20 229 (950)	−667 (1354)	Dominant
10-year time horizon	4.807 (0.053)	4.756 (0.052)	0.051 (0.071)	35 613 (1398)	36 899 (1385)	−1285 (1997)	Dominant
15-year time horizon	6.054 (0.076)	5.978 (0.075)	0.075 (0.106)	48 655 (1570)	50 100 (1535)	−1445 (2216)	Dominant
20-year time horizon	6.830 (0.095)	6.743 (0.085)	0.038 (0.185)	57 988 (1630)	59 835 (1660)	−1846 (2232)	Dominant
25-year time horizon	7.278 (0.102)	7.178 (0.107)	0.100 (0.141)	64 561 (1721)	66 289 (1736)	−1728 (2455)	Dominant
30-year time horizon	7.493 (0.101)	7.388 (0.114)	0.105 (0.155)	68 067 (1830)	70 162 (1898)	−2095 (2608)	Dominant
0% HbA_1c_ change applied for lispro	7.567 (0.127)	7.497 (0.107)	0.071 (0.168)	70 891 (1708)	72 529 (1793)	−1638 (2414)	Dominant
−0.2% HbA_1c_ change applied for lispro	7.661 (0.121)	7.497 (0.107)	0.165 (0.159)	70 735 (1842)	72 529 (1793)	−1794 (2529)	Dominant
0% discount rate	10.981 (0.207)	10.808 (0.192)	0.172 (0.273)	112 330 (2988)	115 059 (2919)	−2729 (4074)	Dominant
7% discount rate	5.688 (0.075)	5.618 (0.069)	0.071 (0.100)	48 988 (1304)	50 449 (1345)	−1461 (1899)	Dominant
Baseline HbA_1c_ of 6.3%	9.323 (0.137)	9.196 (0.14)	0.127 (0.197)	68 434 (2230)	70 384 (2223)	−1949 (3137)	Dominant
54 IU/day for all of insulin treatments	7.601 (0.117)	7.497 (0.107)	0.105 (0.154)	75 511 (1789)	77 345 (1807)	−1835 (2525)	Dominant
54 IU/day for lispro	7.601 (0.117)	7.497 (0.107)	0.105 (0.154)	72 497 (1779)	72 529 (1793)	−32 (2511)	Dominant
54 IU/day for regular insulin	7.601 (0.117)	7.497 (0.107)	0.105 (0.154)	70 576 (1774)	74 346 (1798)	−3770 (2511)	Dominant
No difference in severe hypoglycaemia	7.601 (0.117)	7.567 (0.127)	0.034 (0.159)	70 576 (1774)	70 749 (1708)	−173 (2465)	Dominant
With minor hypoglycaemic event rates applied	3.673 (0.062)	3.318 (0.052)	0.355 (0.079)	70 576 (1774)	72 529 (1793)	−1953 (2508)	Dominant

HbA_1c_, glycated haemoglobin; ICER, incremental cost-effectiveness ratio; QALYs, quality-adjusted life years.

Other sensitivity analyses indicated that the lispro treatment regimen remained dominant at shorter time horizons, even although the magnitude of clinical and cost benefits was reduced. Variation in discount rates had little influence on the overall conclusions from the analysis. Varying the HbA_1c_ benefit associated with lispro between the 95% confidence intervals reported in the Cochrane meta-analysis did not change relative outcomes: lispro remained dominant to RHI. Similarly, reducing mean baseline HbA_1c_ in the simulation cohort to 6.3% (base-case value 9.4%) had little impact on the relative results. Varying assumptions around the insulin doses for the calculation of pharmacy costs did not alter the relative outcomes of the base-case analysis. Assuming 54 IU per day in all insulins (total daily dose of 108 IU for each basal–bolus regimen), the lispro regimen remained cost saving by approximately £1835 vs. RHI over patients’ lifetimes. Increasing the daily dose of only lispro to 54 IU notably reduced the cost saving to only £32 per patient (cost neutral). Conversely, increasing the daily dose of RHI to 54 IU increased the cost saving with lispro to £3770.

## Discussion

In the present study, we conducted a literature review and performed a modelling analysis designed to estimate the long-term implications of basal–bolus therapy with insulin lispro vs. RHI in a population representative of T1DM patients in the UK. The literature review indicated that SAIAs, such as lispro, are associated with fewer postprandial glycaemic excursions, small improvements in HbA_1c_ and notable benefits in terms of hypoglycaemia compared with RHI. Based on these short-term findings, long-term projections using a previously validated model of diabetes indicated that, compared with mealtime RHI, mealtime insulin lispro dominates (more effective and less costly) where the majority of the plotted cost-effectiveness ratios are situated in the south-east quadrant of the plane. Furthermore, the cost-effectiveness acceptability curve showed that there was an estimated probability of 83.9% that lispro will be considered cost-effective at a willingness-to-pay threshold of £30 000. Mealtime insulin lispro is likely to improve life expectancy marginally and quality-adjusted life expectancy, and reduced complication rates and direct medical costs when used as part of a basal–bolus regimen in the UK. These findings are based on the most appropriate data currently available. Sensitivity analysis suggested that these conclusions were robust across variation in a number of key input parameters, including HbA_1c_ change, baseline HbA_1c_ and insulin doses (assuming comparable efficacy). Severe hypoglycaemic event rates were a key driver of outcomes. However, even conservatively assuming no benefit in terms of hypoglycaemia with the insulin lispro regimen, mealtime lispro was still projected to improve quality-adjusted life expectancy and reduce costs in UK patients with T1DM vs. RHI. When considering NICE’s cost-effectiveness threshold range of £20 000–£30 000 per QALY gained, mealtime insulin lispro in combination with basal insulin is likely to be considered an attractive therapy, where the projected ICER for lispro therapy was better than NICE’s acceptable threshold.

A potential shortcoming of the present analysis lies in the inherent uncertainty in making long-term projections based on short-term trial data. We attempted to minimize this uncertainty as far as possible by (i) selecting treatment effect data from a meta-analysis to avoid any bias from one particular study and (ii) using a model of T1DM that has been externally validated against real-world clinical and epidemiological data. Whilst this approach may minimize the uncertainty around the projections reported here, it should be acknowledged that these data are not a substitute for real-life, long-term clinical follow-up data. However, in the absence of long-term trial data, model projections have become an acceptable alternative for a number of health technology assessment bodies around the world [including NICE, CADTH, Scottish Medicines Consortium (SMC) and Pharmaceutical Benefits Advisory Committee (PBAC)].

In the absence of long-term clinical data on the relative effects of mealtime lispro vs. RHI, it was assumed that the clinical benefits on HbA_1c_ and hypoglycaemic event rates would be maintained over the duration of the model simulation (i.e. whilst HbA_1c_ followed a natural creep in both arms, the 0.1%-point benefit with lispro was maintained over the long term). This assumption, that HbA_1c_ benefits can be maintained long term and hypoglycaemic event rates remain relatively constant, are supported by data from the DCCT [34].

The analysis was designed to analyse the long-term outcomes of treatment in a UK-specific T1DM population. On completion of the literature review, it became clear that this created two challenges. The first was that there was no single published data source that provided a complete list of cohort that could be used in the modelling analysis. The cohort used in the simulation is a composite, based on data from THIN database, as this source offered the largest cross-sectional sample of T1DM patients currently published [22]. These data were complemented from other UK-specific registry or database populations rather than clinical trial populations, which are often highly selected. Second, in the modelling simulation, treatment effects used were based on the results of the Cochrane meta-analysis [17]. As such, the treatment effects are based on data from a number of different studies in a number of different populations. Indeed, studies comparing lispro and RHI showed significant heterogeneity (*P* = 0.02). As a result, there is a degree of uncertainty around whether one would expect to see comparable treatment effects on HbA_1c_ and hypoglycaemic event rates in a ‘typical’ UK T1DM population. Importantly, however, one-way sensitivity analysis abolishing the HbA_1c_ benefit or the hypoglycaemia benefit associated with lispro both resulted in lispro remaining dominant to RHI. Moreover, given the conservative approach used in the base-case analysis, whereby minor hypoglycaemic event rates were not incorporated, the present base-case may underestimate the potential benefits of mealtime lispro over RHI in the UK.

## Conclusions

The findings of our literature review indicated that mealtime insulin lispro is associated with short-term benefits in glycaemic control (HbA_1c_ and postprandial glycaemic excursions) and hypoglycaemic event rates compared with mealtime RHI, when both are used as part of a basal–bolus regimen in T1DM. Simulation of long-term outcomes based on these observations, in a population representative of T1DM patients in the UK, indicated that insulin lispro is likely to be associated with improvements in life expectancy, quality-adjusted life expectancy, time to onset of complications, complication rates and lower direct medical costs over patients’ lifetimes compared with RHI.

## Abbreviations

CADTH: Canadian Agency for Drugs and Technologies in Health
CI: confidence interval
CSII: continuous subcutaneous insulin infusion
CVD: cardiovascular disease
DCCT: Diabetes Control and Complications Trial
HbA_1c_: glycated haemoglobin
ICER: incremental cost-effectiveness ratio
IU: International Unit
NICE: National Institute for Health and Clinical Excellence
NPH: neutral protamine Hagedorn
QALE: quality-adjusted life expectancy
QALYs: quality-adjusted life years
RHI: regular human insulin
SAIAs: short-acting insulin analogues
T1DM: Type 1 diabetes mellitus
WMD: weighted mean difference

## Supporting Information

**Appendix S1.** Literature search strategies.

**Appendix S2.** Costs of treatments and complications.
