# Supplementary material for: An estimation of the long-term clinical and economic benefits of insulin lispro in Type 1 diabetes in the UK
Source: Diabet Med. 2009 Aug;26(8):803–14. doi: 10.1111/j.1464-5491.2009.02775.x (PMC3228293; doi:10.1111/j.1464-5491.2009.02775.x)
Supplement: Supplementary file 1 [file dme0026-0803-SD1.doc]

Appendix S1 Literature search strategies

| **Term (insulin)** |  | **Terms (diabetes)** |  | **Terms (UK)** |
| --- | --- | --- | --- | --- |
| insulin analogue*.tw.  *OR*  short acting insulin*.tw.  *OR*  fast acting insulin*.de.  *OR*  rapid acting insulin*.de. | *AND/OR* | diabetes mellitus.tw.  *AND*  type 1.tw. | *AND/OR* | United Kingdom.tw.  *OR*  United Kingdom.de.  *OR*  United Kingdom.ab.  *OR*  United Kingdom.ti,ab. |
| "insulin lispro".de.  *OR*  "insulin aspart".de.  *OR*  "insulin glulisine".de. |  |  |  |  |
| *AND* |  | *AND* |  | *AND* |
| Humalog.ti,ab.  *OR*  Novolog.ti,ab.  *OR*  Apidra.ti,ab.  *OR*  Novorapid.ti,ab. |  | "type 1 diabetes".de.  *OR*  "type 1 diabetes".ab.  *OR*  "type 1 diabetes".tw.  *OR*  "type 1 diabetes".ti,ab. |  | "U.K.".de.  *OR*  "U.K.".ab.  *OR*  "U.K.".tw.  *OR*  "U.K.".ti,ab. |

Search syntax: " "= search phrases; ab = search in article abstract; de = descriptor i.e. subject heading; ti = search in titles; tw = text word

WEB ONLY
