# Supplementary material for: An estimation of the long-term clinical and economic benefits of insulin lispro in Type 1 diabetes in the UK
Source: Diabet Med. 2009 Aug;26(8):803–14. doi: 10.1111/j.1464-5491.2009.02775.x (PMC3228293; doi:10.1111/j.1464-5491.2009.02775.x)
Supplement: Supplementary file 2 [file dme0026-0803-SD2.doc]

Appendix S1 Costs of treatments and complications

| **Description of event or state** | **Annual costs (£)** | **Reference** |
| --- | --- | --- |
| Annual cost of statins | 239.47 | [36] |
| Annual cost of aspirin | 43.1 | [36] |
| Annual cost of angiotensin converting enzyme inhibitor | 60.58 | [36] |
| Annual cost of screening for microalbuminuria | 1.18 | [37] |
| Annual cost of screening for gross proteinuria | 1.18 | [37] |
| Annual cost of eye screening | 31.84 | [38] |
| Monthly cost of non-standard ulcer treatment | 245.6 | [90] |
| Myocardial infarction, year of event | 5228 | [39] |
| Myocardial infarction, each subsequent year | 860.71 | [39] |
| Angina, year of event | 2711.77 | [39] |
| Angina, each subsequent year | 895.96 | [39] |
| Congestive heart failure, year of onset | 3023.31 | [39] |
| Congestive heart failure, each subsequent year | 1059.7 | [39] |
| Stroke, year of event | 3198.41 | [39] |
| Stroke, each subsequent year | 604.88 | [39] |
| Stroke, death within 30 days | 4034.12 | [39] |
| Peripheral vascular disease, year of onset | 2785.68 | [40] |
| Peripheral vascular disease, each subsequent year | 2785.68 | [40] |
| Haemodialysis, first year | 29645.31 | [41] |
| Haemodialysis, each subsequent year | 29645.31 | [41] |
| Peritoneal dialysis, first year | 22259.21 | [41] |
| Peritoneal dialysis, each subsequent year | 22259.21 | [41] |
| Kidney transplant, first year | 23308.74 | [41] |
| Kidney transplant, each subsequent year | 7673.69 | [41] |
| Hypoglycaemic event requiring hospitalization | 444.57 | [42] |
| Ketoacidosis event | 968.73 | [43] |
| Laser treatment for retinal photocoagulation | 897.16 | [41] |
| Cataract operation, year of operation | 1852.19 | [39] |
| Annual cost following cataract operation | 125.07 | [39] |
| Blindness, year of onset | 1039.22 | [39] |
| Annual cost of blindness | 335.42 | [39] |
| Neuropathy, year of onset | 1133.6 | [40] |
| Neuropathy, each subsequent year | 1133.6 | [40] |
| Amputation, year of event | 10461.64 | [39] |
| Amputation, prosthesis | 665.15 | [39] |
| Gangrene treatment | 2455.94 | [44] |
| Cost of infected ulcer treatment | 18351.31 | [44] |
| Cost of uninfected ulcer treatment | 17901.07 | [44] |
| Annual cost after ulcer healed | 0 | [44] |
